# Description of a new species of *Cyrtodactylus* Gray, 1827 (Reptilia, Gekkonidae) from India with redescriptions of the holotypes of *C. gubernatoris* (Annandale, 1913) and *C. himalayicus* (Annandale, 1906)

**DOI:** 10.3897/zookeys.1278.186655

**Published:** 2026-04-30

**Authors:** Sumidh Ray, Bharath Bhupathi, Suvrajyoti Chatterjee, Ritesh Das, Pratyush P. Mohapatra

**Affiliations:** 1 Zoological Survey of India, Reptilia Section, FPS Building, Indian Museum complex, Kolkata 700016, West Bengal, India P.G. Department of Zoology, Fakir Mohan University Balasore India; 2 Freshwater Biology Regional Centre, Zoological Survey of India, Hyderabad 500001, Telangana, India Zoological Survey of India, Reptilia Section, FPS Building, Indian Museum complex Kolkata India; 3 P.G. Department of Zoology, Fakir Mohan University, Balasore 756089, Odisha, India Freshwater Biology Regional Centre, Zoological Survey of India Hyderabad India; 4 Human and Environment Alliance League, 87/20 Bose Pukur Road, Kolkata 700042, West Bengal, India Human and Environment Alliance League Kolkata India

**Keywords:** *Cyrtodactylus peguensis*, Darjeeling, eastern Himalayas, morphology, taxonomy, West Bengal

## Abstract

A new species of the genus *Cyrtodactylus* Gray is described from the eastern Himalayan region of West Bengal state, India. The new species is assigned to the *C. peguensis* species group and is readily distinguished from its regional congeners by a unique combination of morphological characters. These include dorsal scalation comprising small granules intermixed with enlarged, feebly keeled, weakly pointed tubercles arranged in 15–22 fairly regular longitudinal rows at midbody; 11–14 subdigital lamellae beneath the first digit and 17–23 beneath the fourth digit; nine precloacal pores and 6–9 femoral pores in males; 10–12 supralabials; and nine infralabials. Molecular analyses based on mitochondrial NADH dehydrogenase subunit 2 (ND2) sequence data further support the distinctiveness of the new species, which exhibits 11.8–19.8% uncorrected pairwise sequence divergence from its closest congeners. Specifically, it differs from *C. gubernatoris* by 11.8–12.0% and from *C. bhupathyi* by 18.5–19.8%, while showing divergences exceeding 20.5% from other congeners examined. In addition to the description of the new species, detailed redescriptions of the holotypes of two regional congeners, *C. himalayicus* and *C. gubernatoris*, are provided based on direct examination. The discovery of this new taxon highlights the underestimated diversity of *Cyrtodactylus* in the eastern Himalayas and underscores the importance of integrated morphological and molecular approaches in resolving species boundaries within this speciose gecko genus.

## Introduction

*Cyrtodactylus* is among the most speciose genera of geckos (Gekkonidae), currently comprising 398 described species worldwide (Bhattarai et al. 2025a; Uetz et al. 2026), of which 56 are known from India (Mohapatra et al. 2025). The systematics of *Cyrtodactylus* in the Himalayas and adjacent northeast India remained relatively static until the molecular phylogeny by Agarwal et al. (2014), who presented a detailed molecular phylogeny of the *Cyrtodactylus* spp. from Himalayas and Indo-Burma region. Subsequently, a plethora of taxonomic studies have been conducted in this region, resulting in the description of several new species (Agarwal et al. 2018a, 2018b, 2018c; Purkayastha et al. 2020, 2021, 2022; Kamei and Mahony 2021; Mirza et al. 2021, 2022; Bohra et al. 2022; Lalremsanga et al. 2022, 2023; Mahony and Kamei 2022; Boruah et al. 2024; Bharali et al. 2025; Bhardwaj et al. 2025; Bhattarai et al. 2025a, 2025b). Currently this region comprises 45 species among which 19 are distributed in the Himalayas.

In the Himalayas of West Bengal, genus *Cyrtodactylus* is represented by three species, i.e., *C. bhupathyi* Agarwal, Mahony, Giri, Chaitanya & Bauer, *C. gubernatoris* (Annandale) and *C. himalayicus* (Annandale). Agarwal et al. (2014) mentioned the first two species in clade H, and Bhattarai et al. (2025a, 2025b) placed them in *C. peguensis* group (sensu Grismer et al. 2021). The molecular data of *C. himalayicus* cannot be obtained to date. During a recent survey in the Kurseong division of Darjeeling district, we discovered a morphologically and genetically distinct lineage of this group, from Latpanchar, in the outskirts of Mahananda Wildlife Sanctuary. In this paper, we describe this lineage as a new species. Furthermore, we also provide redescriptions of the holotypes of *C. gubernatoris* and *C. himalayicus*.

## Materials and methods

### Sample collection and molecular data acquisition

Specimens were collected by hand from localities outside protected areas in Darjeeling District, West Bengal State, India. Individuals were euthanized following the ethical guidelines for animal euthanasia (Leary et al. 2013). Liver tissue samples were preserved in molecular-grade ethanol (Merck) and stored at -20 °C for genetic analyses. Whole specimens were fixed in 8% formaldehyde by injecting the solution into the body cavity and subsequently soaked overnight. They were then transferred to and stored in 70% ethanol. Voucher specimens were deposited in the collections of the Reptilia Section, Zoological Survey of India, Kolkata, India (**ZSI-R**).

### Morphological and meristic data

Comparative data for the described species of *Cyrtodactylus* from the Himalayas were obtained from published descriptions and comparative accounts of congeners (Agarwal et al. 2018a; Mirza et al. 2022; Bhattarai et al. 2025b). Comparisons were restricted to congeners occurring in the Darjeeling Himalayas, adjacent eastern Nepal and another member of the *C. peguensis* group from the Himalayas. Mensural and meristic data were recorded using a Tomlov digital microscope, primarily on the right side of the body when possible. Photographs were captured with a Nikon ZF camera equipped with a 50-mm macro lens. Colouration was documented from photographs of live specimens. Linear measurements were taken with a Mitutoyo digital vernier calliper to the nearest 0.01 mm and subsequently rounded to the nearest 0.1 mm.

Morphological characters followed Agarwal et al. (2018a), with several additional characters included. The mensural characters recorded were: **SVL**, snout to vent length; **TRL**, trunk length; **BW**, body width; **TL**, tail length; **TW**, tail width; **HL**, head length; **HW**, head width; **HH**, head height; **FL**, forearm length; **CL**, crus length; **OD**, orbital diameter; **NE**, nostril to eye distance; **SE**, snout tip to eye distance; **EE**, eye to ear distance; **EL**, ear length; **IN**, internarial distance; and **IO**, interorbital distance. Meristic characters recorded included: midventral scale rows (**MVSR**, counted between ventrolateral folds); paravertebral tubercles (**PVT**, counted from the most anterior tubercle on the occiput to mid-sacrum); dorsal tubercle rows across the body (**DTR**); supralabials and infralabials (**SL** and **IL**); precloacal pores (**PcP**); femoral pores (**FP**); scales between precloacal and femoral pores; subdigital lamellae under the first and fourth digits of the right manus (**Lam manus I/IV**); and subdigital lamellae under the first and fourth digits of the right pes (**Lam pes I/IV**). In Agarwal et al. (2018a), the last two characters are erroneously labelled as “**R**” (= right) and “**L**” (= left) in Table 3; however, it is correctly described in the ‘Materials and methods’ section as counts for the first and fourth digits.

### Statistical analyses

Morphological data analyses were conducted in R (R Core Team 2021), and graphical outputs were generated using the ggplot2 package (Wickham 2009). Morphometric variation between the new species and its two closest congeners was examined using Principal Component Analysis (**PCA**). Morphometric data for the two closest species were obtained from Agarwal et al. (2018a), assuming the published measurements to be accurate. The PCA incorporated 12 morphometric variables derived from measurements of seven specimens of the new species, including individuals of both sexes. Certain characters were excluded from the analysis if they were damaged in some specimens or showed variation attributable to preservation artifacts. To account for size-related (allometric) effects, morphometric data were adjusted using the GroupStruct package in R (Chan and Grismer 2022), applying allometric scaling following the formulations of Thorpe (1975, 1983). Subsequent multivariate analyses were conducted using the MASS package (Venables and Ripley 2002).

### Molecular data

DNA was extracted using the DNeasy Blood and Tissue Kit (Qiagen, Hilden, Germany). We amplified the complete mitochondrial NADH dehydrogenase subunit 2 (**ND2**) gene using primers L4437 and H5540 (Macey et al. 1997) with up to 1041 nucleotides and sequencing was outsourced at Eurofins India Pvt. Ltd. (Hyderabad, India). The ND2 sequences for the named species were downloaded from GenBank (Table 1). Sequence alignment was performed in MEGA v.12 (Kumar et al. 2024) using default settings. Phylogenetic relationships were analysed using maximum likelihood (**ML**) in IQ-TREE (Nguyen et al. 2015). Uncorrected pairwise sequence divergence was calculated using the pairwise deletion option for members of the genus *Cyrtodactylus* from the Himalayas. Phylogenetic analysis was conducted on the unpartitioned dataset using the GTR + F + I model, with nodal support assessed via 1,000 bootstrap replicates.

**Table 1.** Sequences of the mitochondrial ND2 gene used in this study. Abbreviations for museum and voucher collections are as follows: ZSI, Zoological Survey of India; BNHS, Bombay Natural History Society, Mumbai; CAS, California Academy of Sciences, San Francisco; CES, Centre for Ecological Sciences, Bangalore; LSUHC, La Sierra University Herpetology Collection, Riverside; NHM, Natural History Museum, Kathmandu, Nepal; SB, Santosh Bhattarai field series; WII, Wildlife Institute of India, Dehradun.

Sp. no.	Species	GenBank accession no.	Voucher no.	Locality
1	*Cyrtodactylus nebulicola* sp. nov.	PZ311185	ZSI-R-29064	Latpanchar, Darjeeling district, West Bengal State, India
2	*Cyrtodactylus nebulicola* sp. nov.	PZ311186	ZSI-R-29060	Latpanchar, Darjeeling district, West Bengal State, India
3	*Cyrtodactylus nebulicola* sp. nov.	PZ311187	ZSI-R-29063	Latpanchar, Darjeeling district, West Bengal State, India
4	*Cyrtodactylus gubernatoris*	KM255181	BNHS 2207	Singtam Town, East Sikkim District, Sikkim, India
5	*Cyrtodactylus bhupathyi*	KM255204	CES10/1235	West Bengal State, Darjeeling Dist., Bagdogra, India
6	*Cyrtodactylus tripuraensis*	KM255201	CES10/1210	Rowa Wildlife Sanctuary, North District, Tripura, India
7	*Cyrtodactylus tripuraensis*	KM255182	CES10/1218	Sepahijhala Wildlife Sanctuary, West District, Tripura, India
8	*Cyrtodactylus tripuraensis*	KM255183	BNHS2238	Tripura, India
9	*Cyrtodactylus tripuraensis*	KM255202	CES10/1225	Gumti, North District, Tripura, India
10	*Cyrtodactylus guwahatiensis*	KM255194	BNHS 2146	Assam State, Guwahati Dist., Guwahati, India
11	*Cyrtodactylus jaintiaensis*	KM255195	BNHS 2248	Meghalaya State, Jaintia Hills Dist., near Jowai, India
12	*Cyrtodactylus kazirangaensis*	KM255170	BNHS 2147	Assam State, Golaghat Dist., Kohora, India
13	*Cyrtodactylus kazirangaensis*	KM255188	BNHS 2249	Meghalaya State, East Khasi Hills Dist., Cherrapunjee Resort, Meghalaya, India
14	*Cyrtodactylus montanus*	KM255200	BNHS 2231	Tripura State, North Tripura Dist., Phuldungsei Village, India
15	*Cyrtodactylus nagalandensis*	KM255199	BNHS 2253	Nagaland State, Kohima Dist., Khonoma, India
16	*Cyrtodactylus septentrionalis*	MH971164	BNHS 1989	Assam State, Bongaigaon Dist., near Abhayapuri, India
17	*Cyrtodactylus kamengensis*	OM023869	BNHS 3114	Shergaon, West Kameng District, Arunachal Pradesh, India
18	*Cyrtodactylus kamengensis*	OM023868	BNHS 3113	Shergaon, West Kameng District, Arunachal Pradesh, India
19	*Cyrtodactylus annandalei*	JX440524	CAS215722	Sagaing Div., Mon Ywa Dist., Alaungdaw Kathapa National Park, Myanmar
20	*Cyrtodactylus karanshahi*	PV083873	NHM 2023/373 (SB035)	Nepal, Gandaki Province, Gorkha District, Manaslu Conservation Area, Philim
21	*Cyrtodactylus karanshahi*	PV083872	NHM 2023/372 (SB034)	Nepal, Gandaki Province, Gorkha District, Manaslu Conservation Area, Philim
22	*Cyrtodactylus cf. kamengensis*	KM255196	CES10/1464	Khellong, West Kameng District, Arunachal Pradesh, India
23	*Cyrtodactylus russelli*	JX440555	CAS 226137	Sagaing Div., Htamanthi W.S., Myanmar
24	*Cyrtodactylus pyadalinensis*	MH624105	LSUHC 13932	Shan State, Ywangan Township, Panluang-Pyadalin Cave W.S, Myanmar
25	*Cyrtodactylus nyinyikyawi*	MH624118	CAS 226139	Magwe Reg., Min Bu Township, Shwe Settaw W.S., Myanmar
26	*Cyrtodactylus meersi*	MH624104	LSUHC 13455	Bago Reg., Yangon (north) Dist., Taikkyi Township, Myanmar
27	*Cyrtodactylus peguensis*	MH756190	LSUHC 13454	Bago Reg., Myin Mo Shwe Taung Pagoda, Myanmar
28	*Cyrtodactylus cayuensis*	PQ009369	WII-ADR473	Pasighat, East Siang District, Arunachal Pradesh, India
29	*Cyrtodactylus cayuensis*	PQ009368	WII-ADR454	Jengging, Upper Siang District, Arunachal Pradesh, India
30	*Cyrtodactylus cayuensis*	PQ009367	WII-ADR3016	Glaw Lake, Kamlang Tiger Reserve, Lohit District, Arunachal Pradesh, India
31	*Cyrtodactylus cayuensis*	PQ009370	WII-ADR697	Potin, Lower Subansiri District, Arunachal Pradesh, India
32	*Cyrtodactylus annapurnaensis*	PV083865	NHM 2023/369 (SB031)	Lwang, Kaski District, Gandaki Province, Nepal
33	*Cyrtodactylus annapurnaensis*	PV083864	NHM 2023/368 (SB030)	Lwang, Kaski District, Gandaki Province, Nepal
34	*Cyrtodactylus annapurnaensis*	PV083863	NHM 2023/367 (SB029)	Lwang, Kaski District, Gandaki Province, Nepal
35	*Cyrtodactylus chitwanensis*	PV083869	NHM 2023/362 (SB024)	Kabilas, Chitwan District, Bagmati Province, Nepal
36	*Cyrtodactylus chitwanensis*	PV083868	NHM 2023/366 (SB028)	Kabilas, Chitwan District, Bagmati Province, Nepal
37	*Cyrtodactylus chure*	PX115893	NHM 2025/379 (SB001)	Hariharpurgadhi Fort, Sindhuli District, Bagmati Province, Nepal
38	*Cyrtodactylus chure*	PX115892	NHM 2025/380 (SB002)	Hariharpurgadhi Fort, Sindhuli District, Bagmati Province, Nepal
39	*Cyrtodactylus makwanpurgadhiensis*	PX115891	NHM 2025/383 (SB078)	Makwanpurgadhi Fort, Sindhuli District, Bagmati Province, Nepal
40	*Cyrtodactylus makwanpurgadhiensis*	PX115890	NHM 2025/384 (SB079)	Makwanpurgadhi Fort, Sindhuli District, Bagmati Province, Nepal
41	*Cyrtodactylus makwanpurgadhiensis*	PX115889	NHM 2025/385 (SB080)	Makwanpurgadhi Fort, Sindhuli District, Bagmati Province, Nepal
42	*Cyrtodactylus martinstolli*	PV083875	NHM 2023/359 (SB017)	Dobate, Ilam District, Koshi Province, Nepal
43	*Cyrtodactylus martinstolli*	PV083874	NHM 2023/356 (SB014)	Dobate, Ilam District, Koshi Province, Nepal
44	*Cyrtodactylus nepalensis*	PV083876	NHM 2023/360 (SB038)	Sakayal, Dadeldhura District, Sudurpaschim Province, Nepal
45	*Cyrtodactylus nepalensis*	PV083877	NHM 2023/361 (SB039)	Sakayal, Dadeldhura District, Sudurpaschim Province, Nepal
46	*Cyrtodactylus siangensis*	PQ009372	WII-ADR1582	Bodak, East Siang District, Arunachal Pradesh, India
47	*Cyrtodactylus siangensis*	PQ009371	WII-ADR1581	Bodak, East Siang District, Arunachal Pradesh, India
48	*Cyrtodactylus fasciolatus*	KM255184	CES11/1337	near Subathu, Shimla Dist, Himachal Pradesh State, India

## Results

### Phylogenetic analyses

**Figure 1.**
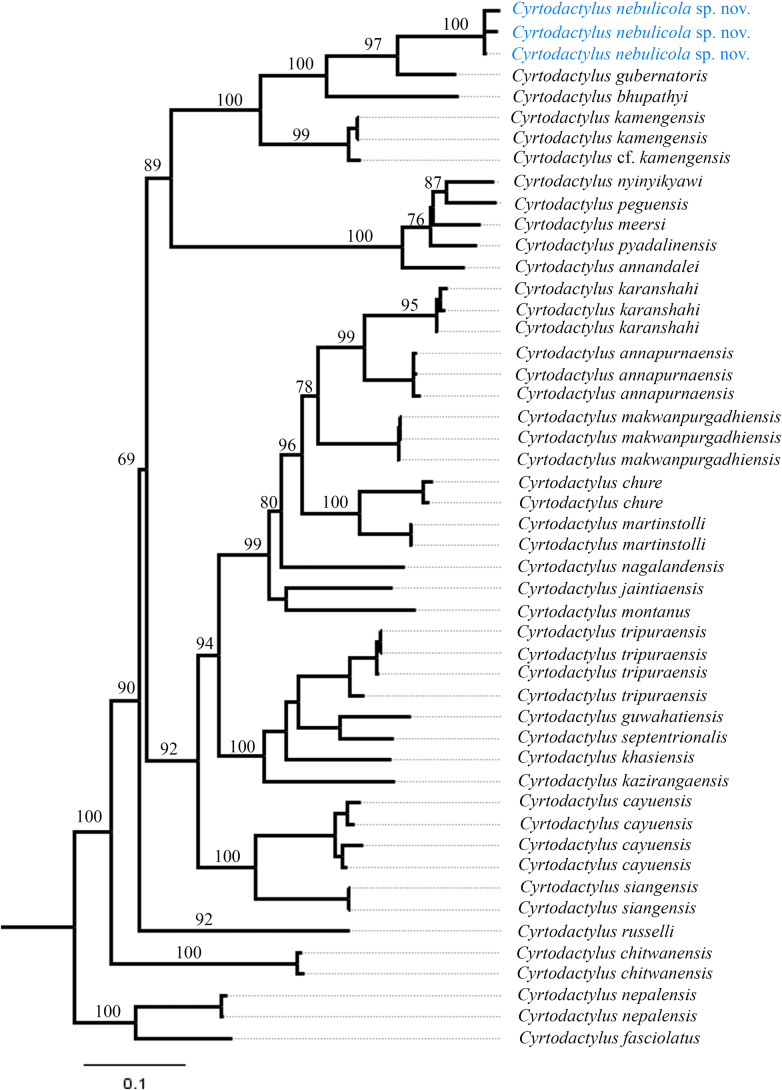
Maximum-likelihood consensus tree based on mitochondrial ND2 gene. Numbers by the nodes indicate ML bootstrap support values.

Phylogenetic analyses recovered a distinct lineage within the *C. peguensis* group (Fig. 1; BS > 98). Genetic analysis based on uncorrected ND2 p-distances indicates that the new lineage differs from its closely related species: *C. gubernatoris* (11.8–12%) and *C. bhupathyi* (18.5–19.8%), and from other congeners by more than 20.5% (Table 2). Based on its phylogenetic position and supported by the genetic divergence, we herein describe this lineage as a new species.

**Table 2.** Pairwise uncorrected ND2 sequence divergence (p-distances) in *Cyrtodactylus* spp. (values in percentage).

Sp. No.	Species	1	2	3	4	5	6	7
1	*C. nebulicola* sp. nov.	**1–3**						
2	*C. gubernatoris*	11.8–12	0.0					
3	*C. bhupathyi*	18.5–19.8	**16**	0.0				
4	*C. kamengensis*	20.5–21.5	19.5	18.5	0.0			
5	*C. cf. kamengensis*	21.5–22.5	19.5	19.5	24.5	0.0		
6	*C. peguensis* group	30–31.5	19.5–28.5	18.5–29.5	24–27.5	24–27	**0.5–26.5**	
7	*C. khasiensis* group	23.5–28.5	22.5–27	23–27.5	22.5–26	22.5–26.5	22.5–30	**0–29**
8	*C. fasciolatus* group	28–29	24–25.5	24.5–26.5	22.5–24	23–25	22–29	15–26.5

**Table 3.** Morphological and meristic data for type specimens of *Cyrtodactylus nebulicola* sp. nov. * denotes damaged body parts.

Voucher No.	ZSI-R-29064 (holotype)	ZSI-R-29060 (paratype)	ZSI-R-29061 (paratype)	ZSI-R-29062 (paratype)	ZSI-R-29063 (paratype)	ZSI-R-29065 (paratype)	ZSI-R-29066 (paratype)
Sex	male	female	female	female	male	female	female
SVL	61.9	75.0	68.2	75.1	71.5	70.0	59.8
TRL	26.8	33.4	29.4	33.3	31.2	33.3	26.7
TRL	26.8	33.4	29.4	33.3	31.2	33.3	26.7
BW	11.5	13.5	12.7	14.8	13.6	11.7	11.7
TL	74.1	*	*	*	*	80.2	*
TW	6.5	6.5	7.4	7.4	8.5	8.0	6.6
HL	17.9	19.0	18.4	19.0	20.6	18.7	16.3
HW	11.9	13.4	12.1	12.7	12.9	12.5	10.8
HH	6.5	7.9	8.1	10.2	7.4	7.1	6.8
FL	8.2	9.0	9.8	10.1	8.9	9.2	8.8
CL	10.0	11.1	11.3	13.2	10.7	10.6	9.8
OD	3.7	4.5	3.1	3.7	4.6	4.0	3.5
NE	5.3	6.0	5.8	5.7	6.5	5.6	5.4
SE	7.1	8.1	8.6	8.0	8.7	7.2	6.5
EE	4.9	5.4	5.4	6.1	5.4	5.5	5.1
EL	1.1	1.4	1.9	1.5	1.5	1.2	1.4
IN	2.5	2.6	2.7	2.7	2.9	2.5	2.3
IO	4.8	6.4	5.3	6.4	5.2	5.2	5.6
FP (L/R)	6/7	5*/5*	*	8/9	7/7	9/7	7/8
PcP	9	9	9	9	9	9	9
MVSR	26	26	26	23	22	23	26
PVT	53	47	49	53	56	51	55
DTR	17	18	16	20	19	15	22
SL (L/R)	10/11	10/10	12/10	10/10	11/11	10/11	10/11
IL (L/R)	9/9	9/9	9/9	9/9	9/9	9/9	9/9
Lam Manus (I/IV)	13/19	11/18	13/19	13/19	13/18	13/19	13/17
Lam Pes (I/IV)	14/21	12/21	13/20	14/21	13/21	14/23	14/22

### Taxonomic account

#### Cyrtodactylus nebulicola sp. nov.

Taxon classificationAnimaliaSquamataGekkonidae

5B8F60AA-5371-551C-8DFB-29302A63E2D8

https://zoobank.org/DAF74733-A089-438B-9828-45FECCA65B3F

**Figure 2.**
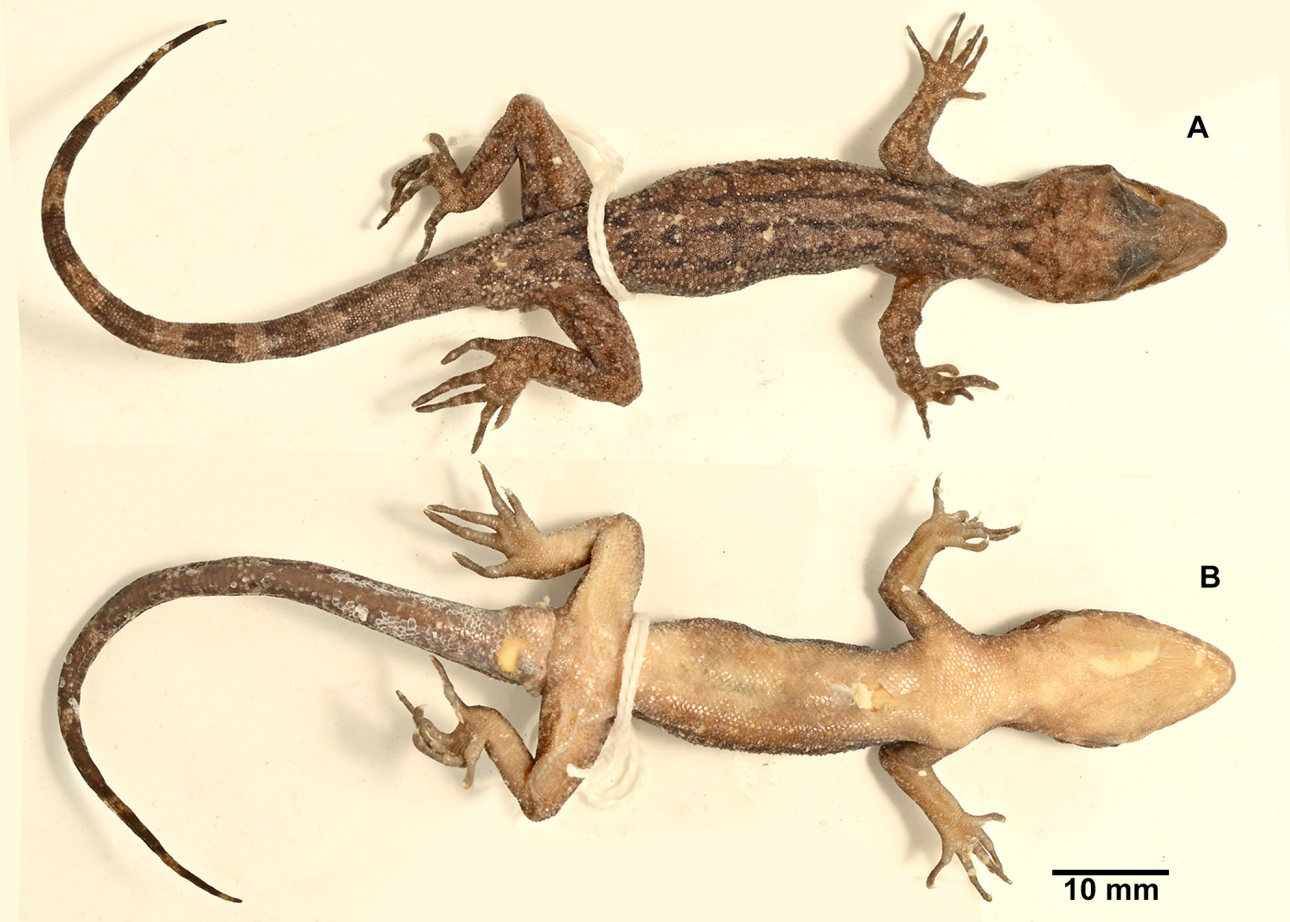
*Cyrtodactylus nebulicola* sp. nov., holotype, ZSI-R-29064. **A**. Dorsal view of body; **B**. ventral view of body.

**Figure 3.**
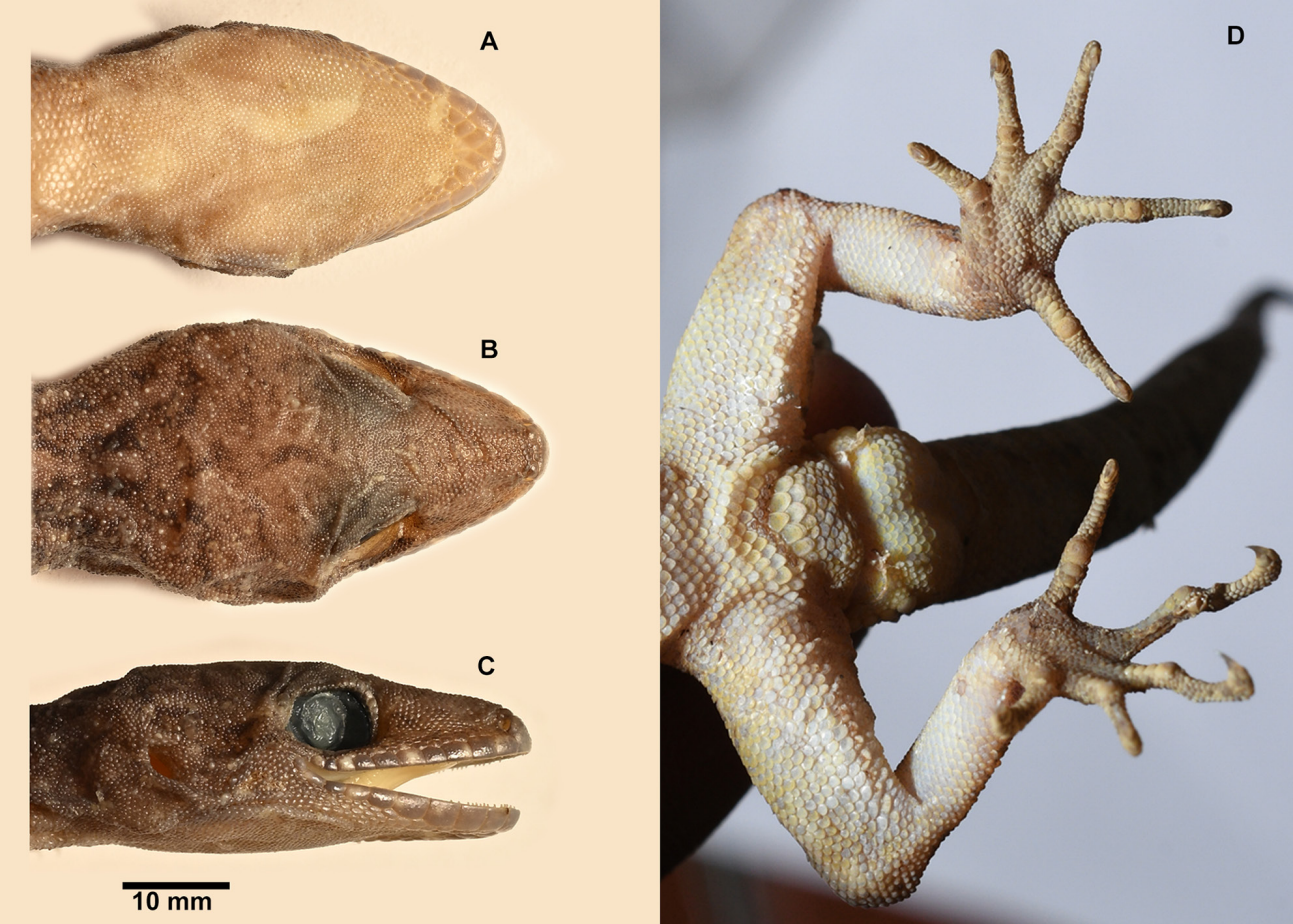
*Cyrtodactylus nebulicola* sp. nov., holotype, ZSI-R-29064. **A**. Ventral view of head; **B**. Dorsal view of head; **C**. Lateral view of head; **D**. View of cloacal region showing pores and digits.

**Figure 4.**
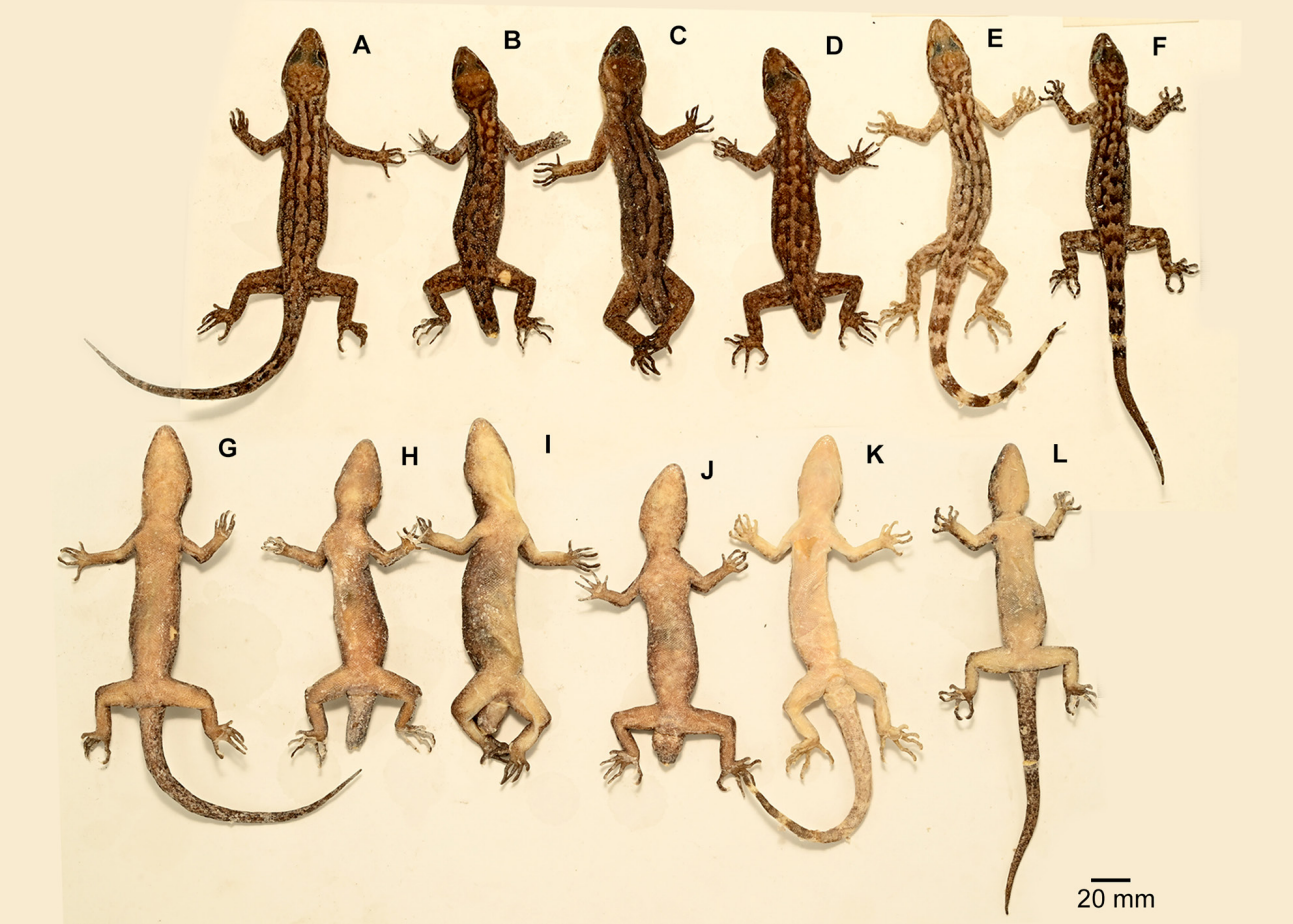
Full body view of all the paratypes of *Cyrtodactylus nebulicola* sp. nov. **A–F**. Dorsal; **G–L**. Ventral; **A, G**. ZSI-R-29060; **B, H**. ZSI-R-29061; **C, I**. ZSI-R-29062; **D, J**. ZSI-R-29063; **E, K**. ZSI-R-29065; **F, L**. ZSI-R-29066.

**Figure 5.**
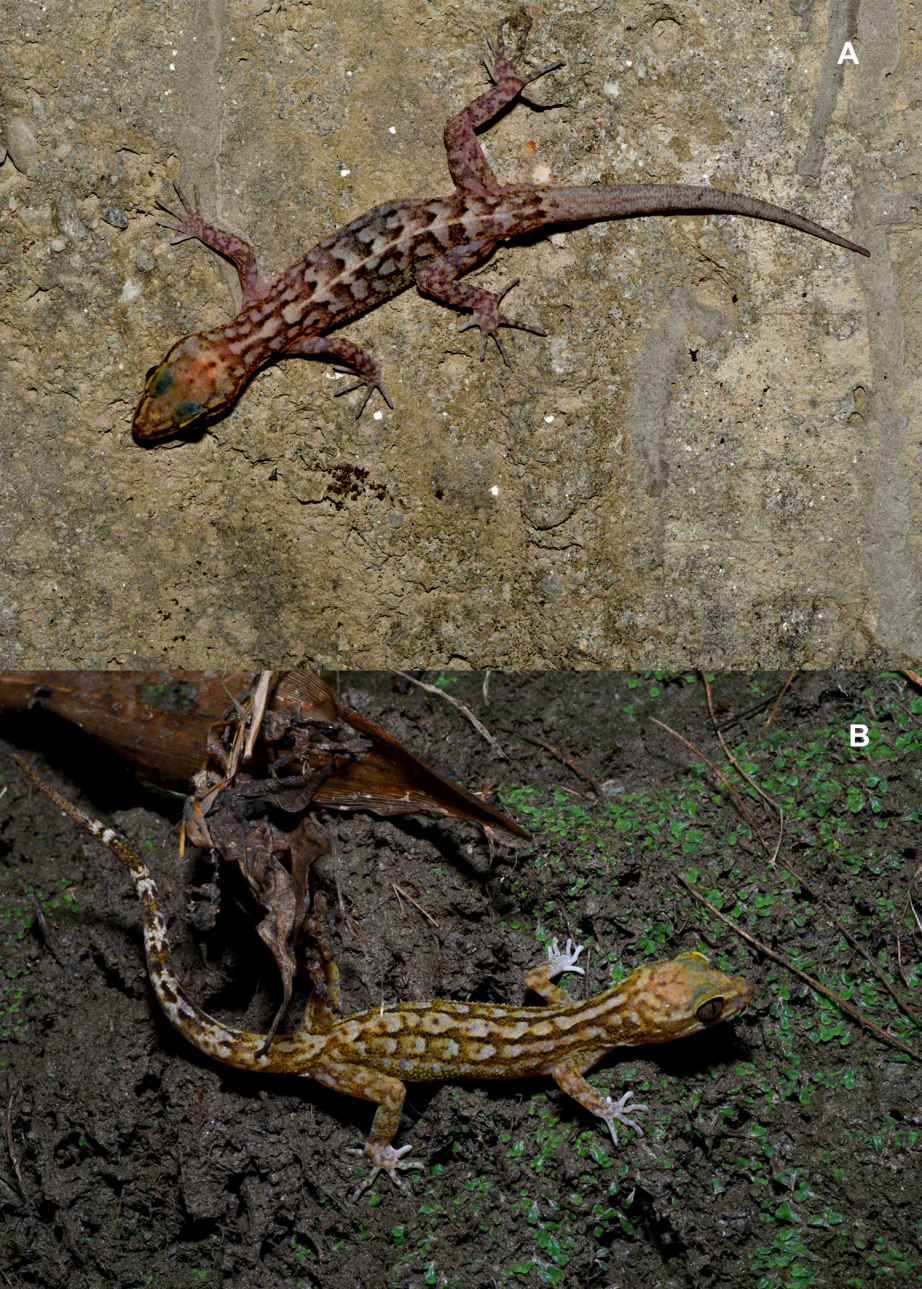
Live specimens of *Cyrtodactylus nebulicola* sp. nov. (A) uncollected individual, (B) one of the paratypes (ZSI-R-29060).

**Figure 6.**
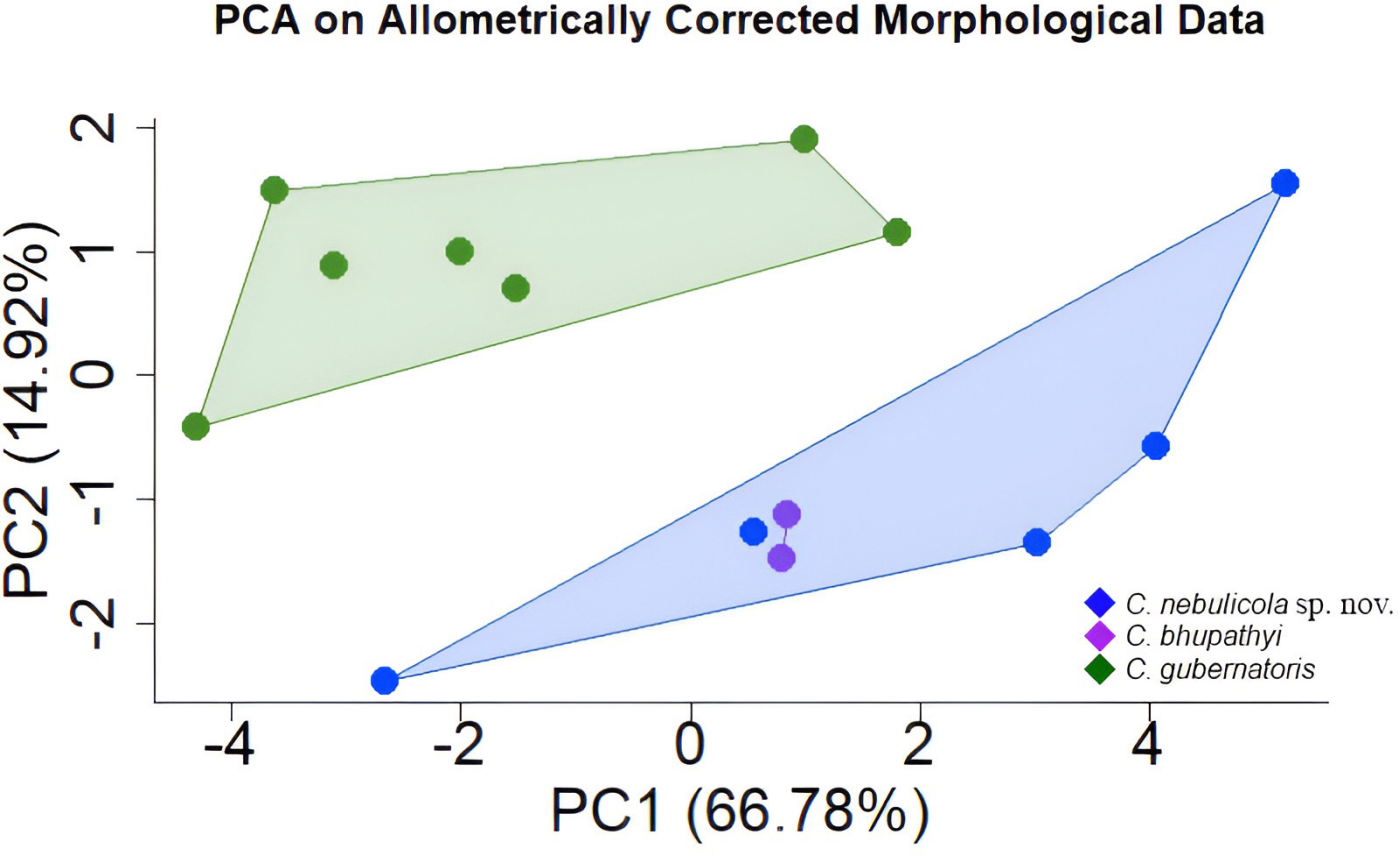
Principal Component Analysis results showing clear separation in morphometric characters between the three closely allied species of *Cyrtodactylus* found in the Eastern Himalayas of Sikkim and West Bengal state, India.

Figs 1, 2, 3, 4, 5, 6

##### Type material.

***Holotype***. (Figs 2, 3) • ZSI-R-29064, adult male, collected by Pratyush P. Mohapatra and Bharath Bhupathi on 17 April 2024, in Latpanchar village (26.9159N, 88.4028E; ca 1100 m a.s.l.), Darjeeling district, West Bengal State, India. ***Paratypes***. (*n* = 6, Fig. 4) • ZSI-R-29060–62, 29065, 29066, adult females, and ZSI-R-29063 adult male, same collection data as holotype.

##### Diagnosis.

A medium-sized *Cyrtodactylus*, reaching a maximum snout–vent length of 75.1 mm (*n* = 7). Dorsal pholidosis heterogeneous, comprising smooth granular scales intermixed with fairly regular rows of enlarged, feebly keeled, weakly pointed tubercles. A ventrolateral fold present along the lower flank. 15–22 longitudinal rows of dorsal tubercles at midbody and 47–56 tubercles in the paravertebral rows. Ventral scales subequal from chest to vent, smooth, rhomboid, and subimbricate with rounded posterior margins; 22–26 scales across the belly at midbody. Subdigital lamellae smooth, mostly entire, with some divided; 13 lamellae (rarely 11; *n* = 1/7) under digit I of the manus and 12–14 under digit I of the pes; 17–19 lamellae under digit IV of the manus and 20–23 under digit IV of the pes. Both sexes possess nine precloacal pores. Males have six or seven femoral pores, whereas females exhibit 7–9 (in one specimen five, with adjacent scales damaged) undeveloped femoral pores (or pitted scales). Scales on the non-regenerated tail dorsum are homogeneous, arranged in fairly regular rows of smooth, flattened, subimbricate scales that are larger than the granular dorsal body scales; enlarged tubercles occur at the tail base. Dorsum patterned with three dark longitudinal streaks from neck to tail base; the original tail bears nine alternating dark and pale bands.

##### Description of holotype.

Adult male in good state of preservation except tail tip curved towards left, a 5.0 mm long incision in sternal region for tissue collection. SVL 61.9 mm, head short (HL/SVL 0.29), wide (HW/HL 0.66), not strongly depressed (HH/HL 0.36), distinct from neck. Loreal region inflated. Snout less than half of head length (SE/HL 0.40), ~ 2× eye diameter (SE/OD 1.92); scales on snout and canthus rostralis smooth, circular, subequal, larger than those on interorbital region; scales on interorbital, occipital, and temporal regions heterogeneous, composed of granular scales intermixed with enlarged, feebly keeled, rounded tubercles. Eye small (OD/HL 0.21); 28 interorbital scale rows across narrowest point of frontal; 43 scale rows between left and right supraciliaries at mid-orbit. Ear-opening small, oval, deep (EL/HL 0.06); eye to ear distance much greater than diameter of eye (EE/OD 1.32). Rostral wider (2.5 mm) than high (1.6 mm), incompletely divided dorsally by a strongly developed rostral groove for almost half of its height; a single enlarged, roughly rectangular supranasal on each side, separated from each other behind rostral by two much smaller internasal scales; rostral in contact with supralabial I, nostril and supranasal, and a single internasal on either side; nostrils oval; two rows of scales separate orbit from supralabials. Mental enlarged, subtriangular, wider (2.6 mm) than high (1.6 mm); three pairs of postmentals, 1^st^ postmental slightly longer (1.7 mm) than mental, in strong contact with each other below mental (1.0 mm); 1^st^ pair bordered by mental, infralabial I, 2^nd^ postmental either side and additionally by four slightly enlarged gular scales; 2^nd^ postmentals smaller (1.1 mm) than the 1^st^ pair, bordered by 1^st^ postmentals, infralabial I on right side and I & II on left, 3^rd^ postmentals, and four gular scales on right side and three on left; 3^rd^ postmentals smaller (0.8 mm) than the 2^nd^ pair, bordered by 2^nd^ postmentals, infralabial I & II on right side and II on left, and three gular scales on each side; all gular scales bordering postmentals subequal, subcircular, smooth, and much smaller than postmentals; scales on rest of throat, granular, much smaller, smooth, and subcircular. Infralabials bordered below by two rows of slightly enlarged, much elongated scales, decreasing in size posteriorly. Eleven supralabials on right side and ten on left to angle of jaw; nine infralabials to angle of jaw on either side.

Body relatively slender (BW/TRL 0.43), trunk just less than half of SVL (TRL/SVL 0.43) with ventrolateral fold of skin on lower flank. Dorsal pholidosis heterogeneous; smooth granular scales intermixed with somewhat regularly arranged rows of enlarged, feebly keeled, weakly pointed tubercles; granular scales slightly larger in the flank region than the paravertebral region; granular scales on occiput slightly smaller than paravertebral granular scales; enlarged tubercles in ~ 17 longitudinal rows at midbody; 53 tubercles in paravertebral row. Ventral scales much larger than granular scales on dorsum, subequal from chest to vent, and smooth, rhomboid and subimbricate with rounded end; midbody scale rows across belly 26. Nine precloacal pores; seven femoral pores on the right side and six on the left. Scales on palm and soles, smooth, heterogeneous in size and shape; scales on dorsal aspects of limbs heterogenous; upper arm with slightly smaller scales than body ventrals, smooth and subimbricate; scales on lower arm composed of slightly smaller, smooth, granular scales intermixed with enlarged, smooth, rounded, weakly pointed tubercles; thigh and crus with slightly smaller, weakly keeled, granular scales intermixed with enlarged, smooth, rounded, weakly pointed tubercles; scales on ventral aspect of upper arm smooth, granular, slightly smaller than granular scales on body dorsum, scales on ventral aspect of lower arm subequal with those on upper arm, smooth, subcircular, weakly conical to flattened; ventral aspect of thigh and crus with enlarged, smooth, subcircular, flattened, subimbricate scales; scales on precloacal region and pore bearing femoral scale row distinctly enlarged. Forelimbs and hindlimbs slightly long, slender (FL/ SVL 0.13; CL/SVL 0.16). Digits with unpaired lamellae, separated into a basal and narrower distal series by a single, much enlarged lamella at inflection; lamellae (on right side) on manus: (13-13-18-19-14), and on pes: (14-17-19-21-17). Relative length of digits (measurements in mm in parentheses): III (5.7) > IV (5.6) > II (5.5) > V (4.9) > I (3.7) (right manus); IV (7.3) > III (6.9) > V (6.4) > II (6.2) > I (3.9) (right pes). Tail original, subcylindrical, slender, entire, slightly longer than body (TL/SVL 1.13). Dorsal pholidosis on tail homogeneous; composed of fairly regularly arranged, smooth, subcircular, flattened, and subimbricate scales that are larger than granular scales on midbody dorsum, gradually becoming larger posteriorly and dorsolaterally; enlarged tubercles present on the tail base. Scales on tail venter much larger than those on dorsal aspect, smooth, flattened, subimbricate. Scales on ventral aspect of tail base much smaller, smooth, subimbricate; distinct hemipenial bulge present, part of the skin damaged; four subequal and smooth postcloacal spurs on either side.

##### Colouration (Fig. 5).

Dorsum pale brown to greyish-brown. Head slightly paler than trunk, with irregular darker brown mottling and faint reticulations on crown and snout; temporal region with indistinct dark patches. A poorly defined mid-dorsal pattern present, composed of irregular, fused dark brown blotches and short longitudinal streaks extending from nape to sacral region; dorsolateral areas with scattered dark brown marbling and transverse bars. Limbs pale brown, marked with irregular dark brown spots and short bands; digits paler distally, beige to off-white, digital pads paler than limb bases. Tail distinctly annulated, bearing alternating dark brown and pale cream to beige bands; dark bands broader and more conspicuous proximally, becoming narrower and less distinct towards distal portion, a total of nine dark bands present; tail tip dusky brown. Ventral surfaces uniformly pale cream to off-white, largely immaculate, lacking distinct markings. Overall colouration cryptic and disruptive, suited for camouflage against rocky or bark substrates.

##### Variations.

Mensural and meristic data for the type series are presented in Table 3. Two paratypes (ZSI-R-29060 and ZSI-R-29066) possess regenerated tails with broken tips. Additionally, two female paratypes (ZSI-R-29060 and ZSI-R-29061) exhibit damaged femoral scales.

##### Multivariate analyses.

Principal Component Analysis (PCA) was employed to examine morphometric variation among three closely related *Cyrtodactylus* lineages from India, using a dataset comprising 12 morphometric variables (Suppl. material 1). The distribution of specimens in multivariate morphospace was visualised to assess lineage-level differentiation (Fig. 6). The first two principal components together accounted for 81.7% of the total observed variance. Factor loadings associated with each morphometric variable are provided in Table 4.

**Table 4.** PCA Factor loadings.

	PC1	PC2	PC3	PC4	PC5	PC6
SVL	0.33516	−0.05310	0.04973	0.21993	0.07130	−0.13290
TRL	0.30985	0.07641	−0.12470	0.41882	0.51197	−0.21390
HL	0.32091	−0.22560	−0.01440	0.04010	0.27643	0.12802
HW	0.31254	−0.21700	0.15359	0.33438	0.00175	−0.06880
HH	0.28059	0.14604	0.46667	0.10843	−0.34330	−0.29770
FL	0.23939	0.52027	−0.20200	−0.29970	0.35426	0.23077
CL	0.25902	0.51668	−0.08250	−0.13730	−0.23880	−0.27180
OD	0.17929	−0.19480	−0.76470	0.15823	−0.46510	−0.10680
NE	0.29509	−0.29640	−0.18170	−0.37110	0.12539	0.13747
SE	0.29980	−0.16270	0.07468	−0.56920	−0.02670	−0.42210
EE	0.30018	0.31070	0.02529	0.17868	−0.30160	0.54193
IN	0.29774	−0.29250	0.26235	−0.15390	−0.17970	0.44453

##### Distribution and natural history.

*Cyrtodactylus nebulicola* sp. nov. is currently known only from its type locality in Latpanchar, Darjeeling district, West Bengal State, India. The species inhabits in the rock crevices beside the village roads as well as house walls and the area is surrounded by the dense mixed-wet forest. Within its distributional range, the species occurs sympatrically with other lizards, including *Hemidactylus platyurus* (Schneider) of family Gekkonidae, *Japalura variegata* Gray of family Agamidae, and *Ablepharus sikimmensis* (Blyth) and *Sphenomorphus maculatus* (Blyth) of family Scincidae.

##### Comparison with regional congeners.

The new species was phylogenetically recovered as part of the *Cyrtodactylus peguensis* group. This group comprises only three species in the Himalayas, including *C. bhupathyi*, *C. gubernatoris* and *C. kamengensis* Mirza, Bhosale, Thackeray, Phansalkar, Sawant, Gowande & Patel. We therefore restrict comparisons to these three species along with two other regional congeners, namely *C. himalayicus* from Darjeeling district and *C. martinstolli* (Darevsky, Helfenberger, Orlov & Shah) from adjacent eastern Nepal. *C. nebulicola* sp. nov. can be distinguished from the aforementioned congeners by the following com­bination of morphological characters: 15–22 DTR (vs 24–25 in *C. bhupathyi*, 20–24 in *C. kamengensis*, 19–23 in *C. martinstolli*); 6–9 FP (vs 4–7 in *C. bhupathyi*, absent in *C. himalayicus*, *C. kamengensis* and *C. martinstolli*); FP present in females (vs absent in *C. gubernatoris*, *C. himalayicus*, *C. kamengensis* and *C. martinstolli*); PcP 9 (vs 10–11 in *C. bhupathyi*, 10 in *C. himalayicus*); MVSR 22–26 (vs 37–38 in *C. bhupathyi*, 34–37 in *C. gubernatoris*, 33–34 in *C. himalayicus*, 30–34 in *C. kamengensis*, 35–40 in *C. martinstolli*); PVT 47–56 (vs 36–45 in *C. gubernatoris*, 49–58 in *C. kamengensis*, 30–37 in *C. martinstolli*); median subcaudal scales not enlarged (vs enlarged in *C. bhupathyi* and *C. kamengensis*); maximum SVL 75.1 (vs 61.5 in *C. bhupathyi*, 70.5 in *C. gubernatoris*, 64.5 in *C. himalayicus*, 78.6 in *C. kamengensis*, 80.4 in *C. martinstolli*); ventrolateral fold present (vs absent in *C. himalayicus* and *C. martinstolli*); subdigital lamellae under first digit of manus 11–13 (vs 10–19 in *C. gubernatoris*, 10–11 in *C. kamengensis*, 14–15 in *C. martinstolli*), under fourth digit of manus 17–19 (vs 15–23 in *C. gubernatoris*, 15–18 in *C. kamengensis*, 18–20 in *C. martinstolli*), under first digit of pes 12–14 (vs 12–15 in *C. bhupathyi*, 12–17 in *C. gubernatoris*, 11–13 in *C. kamengensis*, 14–15 in *C. martinstolli*), under fourth digit of pes 20–23 (vs 17–18 in in *C. bhupathyi*, 19–26 in *C. gubernatoris*, 16–21 in *C. kamengensis*, 19–22 in *C. martinstolli*); and by the presence of two internasals (vs one in *C. gubernatoris* and *C. himalayicus*, and one or two in *C. martinstolli*). A detailed comparison of diagnostic characters among these species is provided in Table 5.

**Table 5.** Comparative key morphological characters of *Cyrtodactylus nebulicola* sp. nov. with regional congeners.

Species	DTR	FP	FP in females	PcP	MVSR	PVT	Median suncaudal	Maximum SVL	Ventrolateral fold	Lam manus (I/IV)	Lam pes (I/IV)	Internasal
*C. nebulicola* sp. nov.	15–22	6–9	present	9	22–26	47–56	not enlarged	75.1	present	11–13/17–19	12–14/20–23	2
*C. bhupathyi*	24–25	4–7	present	10–11	37–38	51–55	enlarged	61.5	present	12/18–19	12–15/17–18	2
*C. gubernatoris*	20–21	6–9	absent	7–9	34–37	36–45	not enlarged	70.5	present	10–19/15–23	12–17/19–26	1
*C. himalayicus*	19–21	absent	absent	10	33–34	49	not enlarged	64.5	absent	*	*	1
*C. kamengensis*	20–24	absent	absent	7–11	30–34	49–58	enlarged	78.6	present	10–11/15–18	11–13/16–21	2
*C. martinstolli*	19–23	absent	absent	7–9	35–40	30–37	not enlarged	80.4	present	14–15/18–20	14–15/19–22	1–2

##### Suggested common English name.

Latpanchar bent-toed gecko.

##### Etymology.

The species epithet *nebulicola* is derived from the Latin words: *nebula* meaning “mist” or “cloud,” and -*cola* meaning “dweller” or “inhabitant”, collectively meaning “dweller of the mist”. The name refers to the characteristic mist-laden, cloud-forest habitat of Latpanchar in the Darjeeling Himalaya, where the species was discovered. The epithet is treated as a noun in apposition and does not change with gender.

#### Cyrtodactylus gubernatoris

Taxon classificationAnimaliaSquamataGekkonidae

(Annandale, 1913)

1F0AE586-8965-5F1E-B854-0A2A79760974

Fig. 7

##### Type material examined.

**Figure 7.**
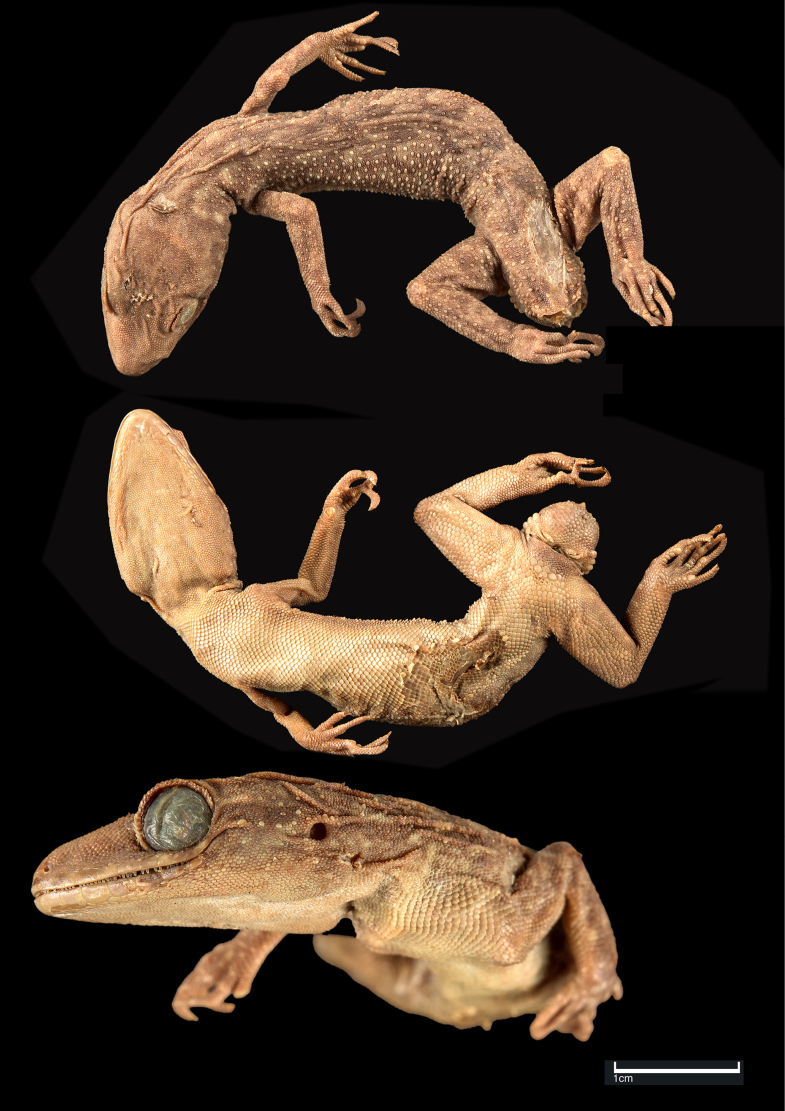
*Cyrtodactylus gubernatoris* (holotype, ZSI-R-17275). Scale bar: 1 cm.

***Holotype***. (Fig. 7) • ZSI-R-17275, adult male, collected by H.E. Lord Carmichael in Darjeeling district, West Bengal State, India.

##### Remarks on ZSI-R-17276.

Das et al. (1998) designated this specimen as a paratype, a treatment subsequently followed by Agarwal et al. (2018a) and Uetz et al. (2026). However, Annandale (1913) explicitly identified only ZSI-R-17275 as a type, and did not assign this status to the other specimen (ZSI-R-17276). Therefore, ZSI-R-17276 should be regarded as a referred specimen rather than a paratype.

##### Redescription of holotype.

Adult male with body curved towards left and broken tail. SVL 51.8 mm, head short (HL/SVL 0.28), wide (HW/HL 0.64), not strongly depressed (HH/HL 0.36), distinct from neck. Loreal region inflated. Snout < 1/2 of head length (SE/HL 0.44), ~ 2× eye diameter (SE/OD 1.94); scales on snout and canthus rostralis granular, subequal, larger than those on interorbital region; scales on interorbital, occipital, and temporal regions heterogeneous, composed of granular scales intermixed with enlarged, feebly keeled, rounded tubercles. Eye small (OD/HL 0.23); 27 interorbital scale rows across narrowest point of frontal; 36 scale rows between left and right supraciliaries at mid-orbit. Ear-opening small, oval, deep (EL/HL 0.05); eye to ear distance much greater than diameter of eye (EE/OD 1.36). Rostral ~ 2× wider (2.5 mm) than high (1.5 mm), incompletely divided dorsally by a strongly developed rostral groove for almost half of its height; a single enlarged, roughly rectangular supranasal on each side, separated from each other behind rostral by a single, much smaller internasal scale; rostral in contact with supralabial I, nostril, supranasal and internasal; nostrils oval; one to two rows of scales separate orbit from supralabials. Mental enlarged, subtriangular, wider (2.1 mm) than high (1.5 mm); three pairs of postmentals, 1^st^ postmental almost as long (1.4 mm) as mental, in strong contact with each other below mental (0.7 mm); 1^st^ pair bordered by mental, infralabial I, 2^nd^ postmental either side and additionally by four slightly enlarged gular scales; 2^nd^ postmentals smaller (0.8 mm) than the 1^st^ pair, bordered by 1^st^ postmentals, infralabials I & II, 3^rd^ postmentals, and two gular scales on each side; 3^rd^ postmentals almost as long (0.8 mm) as 2^nd^ pair, bordered by 2^nd^ postmentals, infralabial II, and four gular scales on right side and two on left; all gular scales bordering postmentals subequal, subcircular, smooth, and much smaller than postmentals; scales on rest of throat, granular, much smaller, smooth, and subcircular. Infralabials bordered below by two rows of slightly enlarged, much elongated scales, decreasing in size posteriorly. Ten supralabials on right side, eleven on left, and ten infralabials on right side and nine on left to the angle of jaw.

Body relatively slender (BW/TRL 0.34), trunk just less than half of SVL (TRL/SVL 0.45) with ventrolateral fold of skin on lower flank. Dorsal pholidosis heterogeneous; smooth granular scales intermixed with somewhat regularly arranged rows of enlarged, feebly keeled, weakly pointed tubercles; granular scales on occiput slightly smaller than paravertebral granular scales; enlarged tubercles in ~ 21 longitudinal rows at midbody; 45 tubercles in paravertebral row. Ventral scales much larger than granular scales on dorsum, subequal from chest to vent, and smooth, roughly pentagonal and subimbricate with rounded end; midbody scale rows across belly 34. Eight precloacal pores; seven femoral pores on the right side and six on the left. Scales on palm and soles, smooth, heterogeneous in size and shape; scales on dorsal aspects of limbs heterogenous; upper arm with slightly smaller scales than body ventrals, smooth and subimbricate; scales on lower arm composed of slightly smaller, smooth, granular scales intermixed with enlarged, smooth, rounded, weakly pointed tubercles; thigh and crus with slightly smaller, weakly keeled, granular scales intermixed with enlarged, smooth, rounded, weakly pointed tubercles; scales on ventral aspect of upper arm smooth, granular, slightly smaller than granular scales on body dorsum, scales on ventral aspect of lower arm subequal with those on upper arm, smooth, subcircular, weakly conical to flattened; ventral aspect of thigh and crus with enlarged, smooth, subcircular, flattened, subimbricate scales; scales on precloacal region and pore bearing femoral scale row not enlarged. Forelimbs and hindlimbs slightly long, slender (FL/ SVL 0.14; CL/SVL 0.18). Digits with unpaired lamellae, separated into a basal and narrower distal series by a single, much enlarged lamella at inflection; lamellae (on right side) on manus: (10-13-17-15-13), and on pes: (12-16-18-19-17). Relative length of digits (measurements in mm in parentheses): IV (4.8) > III (4.7) > II (4.5) = V (4.5) > I (3.7) (right manus); IV (6.2) > V (5.4) > III (5.0) > II (4.6) > I (4.4) (right pes). Tail broken.

#### Cyrtodactylus himalayicus

Taxon classificationAnimaliaSquamataGekkonidae

(Annandale, 1906)

075A4746-8551-5C98-BC3E-6E40788AF119

Fig. 8

##### Type material examined.

**Figure 8.**
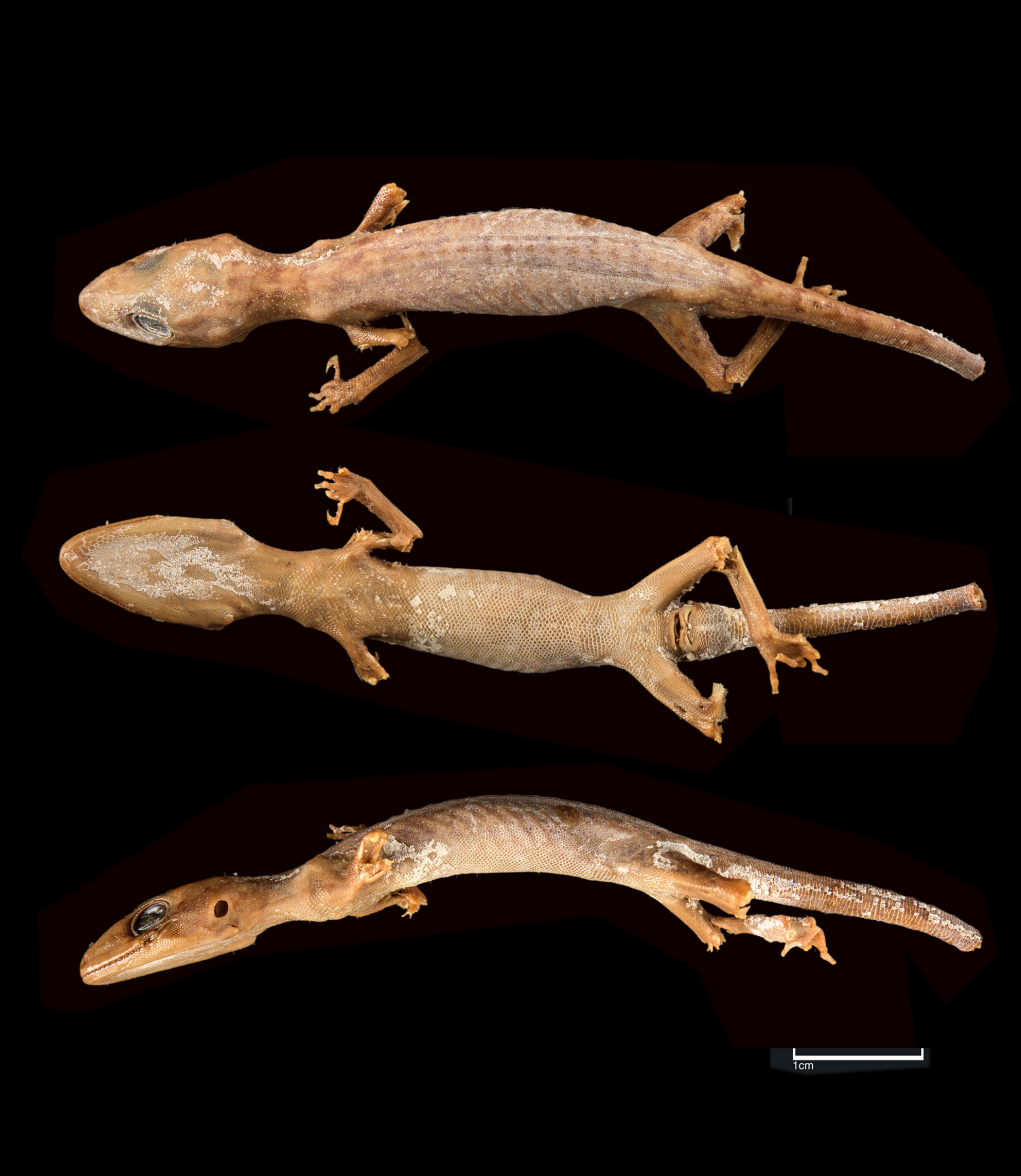
*Cyrtodactylus himalayicus* (holotype, ZSI-R-15716). Scale bar: 1 cm.

***Holotype***. (Fig. 8) • ZSI-R-15716, male, collected by N. Annandale in Kurseong, Darjeeling district, West Bengal State, India.

##### Redescription of holotype.

Male with a detached tail. SVL 50.1 mm, head short (HL/SVL 0.28), wide (HW/HL 0.62), not strongly depressed (HH/HL 0.37), distinct from neck. Loreal region inflated. Snout less than half of head length (SE/HL 0.48), > 2× eye diameter (SE/OD 2.13); scales on snout and canthus rostralis smooth, circular, subequal, larger than those on interorbital region; scales on interorbital, occipital, and temporal regions heterogeneous, composed of granular scales intermixed with enlarged, feebly keeled, rounded tubercles. Eye small (OD/HL 0.22); 22 interorbital scale rows across narrowest point of frontal; 28 scale rows between left and right supraciliaries at mid-orbit. Ear-opening small, oval, deep (EL/HL 0.10); eye to ear distance almost same as the diameter of eye (EE/OD 1.13). Rostral ~ 2× wider (3.4 mm) than high (1.8 mm), undivided; a single enlarged, roughly rectangular supranasal on each side, separated from each other behind rostral by one smaller internasal scale; rostral in contact with supralabial I, nostril and supranasal, and a single internasal on either side; nostrils oval; two rows of scales separate orbit from supralabials. Mental enlarged, subtriangular, wider (2.2 mm) than high (1.5 mm); two pairs of postmentals, 1^st^ postmental as long as (1.5 mm) mental, in strong contact with each other below mental (0.9 mm); 1^st^ pair bordered by mental, infralabial I, 2^nd^ postmental either side and additionally by three slightly enlarged gular scales; 2^nd^ postmentals smaller (0.7 mm) than the 1^st^ pair, bordered by 1^st^ postmentals, infralabial I, and four gular scales on right side and three on left; all gular scales bordering postmentals subequal, subcircular, smooth, and much smaller than postmentals; scales on rest of throat, granular, much smaller, smooth, and subcircular. Infralabials bordered below by two rows of slightly enlarged, much elongated scales, decreasing in size posteriorly. Ten supralabials on the left side to angle of jaw, right side damaged; eight infralabials on the right side and nine on the left to angle of jaw.

Body relatively slender (BW/TRL 0.37), trunk just less than half of SVL (TRL/SVL 0.43). Dorsal pholidosis heterogeneous; smooth granular scales intermixed with somewhat regularly arranged rows of enlarged, feebly keeled, weakly pointed tubercles; granular scales slightly larger in the flank region than the paravertebral region; granular scales on occiput slightly smaller than paravertebral granular scales; enlarged tubercles in ~ 19 longitudinal rows at midbody; 49 tubercles in paravertebral row. Ventral scales much larger than granular scales on dorsum, subequal from chest to vent, and smooth, rhomboid and subimbricate with rounded end; midbody scale rows across belly 34. Eight precloacal pores. Scales on palm and soles, smooth, heterogeneous in size and shape; scales on dorsal aspects of limbs heterogenous; upper arm with slightly smaller scales than body ventrals, smooth and subimbricate; scales on lower arm composed of slightly smaller, smooth, granular scales intermixed with enlarged, smooth, rounded, weakly pointed tubercles; thigh and crus with slightly smaller, weakly keeled, granular scales intermixed with enlarged, smooth, rounded, weakly pointed tubercles; scales on ventral aspect of upper arm smooth, granular, slightly smaller than granular scales on body dorsum, scales on ventral aspect of lower arm subequal with those on upper arm, smooth, subcircular, weakly conical to flattened; ventral aspect of thigh and crus with enlarged, smooth, subcircular, flattened, subimbricate scales; scales on precloacal region enlarged. Forelimbs and hindlimbs slightly long, slender (FL/ SVL 0.14; CL/SVL 0.17). Digits with unpaired lamellae; toe tips broken off. Tail original, detached. Dorsal pholidosis on tail composed of fairly regularly arranged, smooth, subcircular, flattened, and subimbricate scales that are larger than granular scales on midbody dorsum; enlarged tubercles present on the tail base. Scales on tail venter much larger than those on dorsal aspect, smooth, flattened, subimbricate. Scales on ventral aspect of tail base much smaller, smooth, subimbricate; distinct hemipenial bulge present.

## Discussion

*Cyrtodactylus nebulicola* sp. nov. represents the sixth endemic reptile species reported from the state of West Bengal (Mohapatra et al. 2025), following *Cyrtodactylus bhupathyi* and *C. himalayicus* (Gekkonidae), *Indotyphlops meszoelyi* (Wallach) (Typhlopidae), *Gerrhopilus oligolepis* (Wall) (Gerrhopilidae), and *Platyceps vittacaudatus* Kuhl (Colubridae). Despite this notable endemism, the lizard fauna of West Bengal, particularly within the Darjeeling Himalayan region, remains poorly studied. Several unresolved taxonomic issues persist, notably among geckos such as *Hemidactylus platyurus* and *H. cf. brookii* Gray, as well as skinks including *Ablepharus sikimmensis* and *Sphenomorphus maculatus*.

Although local communities have historically contributed to biodiversity conservation, as exemplified by the protection of the Himalayan salamander *Tylototriton verrucosus* Anderson, recent increases in tourism and wildlife photography in rural and montane areas have resulted in escalating anthropogenic disturbance. Habitat degradation and increased human presence may pose significant threats to narrowly distributed and habitat-specialist reptiles such as *C. nebulicola* sp. nov. These findings emphasise the necessity of integrating systematic taxonomic research with habitat protection, community-based conservation initiatives, and regulation of unmonitored tourism to ensure the long-term persistence of herpetofaunal diversity in the Darjeeling Himalayas.

## Supplementary Material

XML Treatment for Cyrtodactylus nebulicola

XML Treatment for Cyrtodactylus gubernatoris

XML Treatment for Cyrtodactylus himalayicus

## Additional information

### Artificial Intelligence (AI) use

Regarding the use of AI in the preparation of this manuscript, the authors declare the following:

Grammarly and other AI tools were used for english editing.

### Author ORCIDs

S. Ray https://orcid.org/0009-0001-2751-5122

B. Bhupathi https://orcid.org/0000-0001-7872-5608

P.P. Mohapatra https://orcid.org/0000-0002-8966-297X

### Data availability

All of the data that support the findings of this study are available in the main text or Supplementary Information.

## Supplementary materials

Supplementary material 1 Morphometric characters Data type docx Explanation note **fig. S1**. Violin plots of morphometric characters of three closely related members of *Cyrtodactylus nebulicola* sp. nov., *C. gubernatoris* (Annandale, 1913) and *C. bhupathyi* Agarwal, Mahony, Giri, Chaitanya & Bauer, 2018. https://binary.pensoft.net/file/1614995This dataset is made available under the Open Database License (http://opendatacommons.org/licenses/odbl/1.0/). The Open Database License (ODbL) is a license agreement intended to allow users to freely share, modify, and use this Dataset while maintaining this same freedom for others, provided that the original source and author(s) are credited. Sumidh Ray, Bharath Bhupathi, Suvrajyoti Chatterjee, Ritesh Das, Pratyush P. Mohapatra

## References

[B1] Agarwal I, Bauer AM, Jackman TR, Karanth KP (2014) Insights into Himalayan biogeography from geckos: A molecular phylogeny of *Cyrtodactylus* (Squamata: Gekkonidae). Molecular Phylogenetics and Evolution 80: 145–155. 10.1016/j.ympev.2014.07.018 25108260

[B2] Agarwal I, Mahony S, Giri VB, Chaitanya R, Bauer AM (2018a) Two new species of bent toed geckos, *Cyrtodactylus* Gray, 1827 (Squamata: Gekkonidae) from Northeast India with comments on name-bearing types from the region. Zootaxa 4420(3): 334–356. 10.11646/zootaxa.4420.3.2 30313531

[B3] Agarwal I, Khandekar A, Bauer AM (2018b) A new bent-toed gecko (Squamata: Gekkonidae: *Cyrtodactylus*) from the Western Himalayas, Himachal Pradesh, India. Zootaxa 4446(4): 442–454. 10.11646/zootaxa.4446.4.2 30313869

[B4] Agarwal I, Mahony S, Giri VB, Chaitanya R, Bauer AM (2018c) Six new *Cyrtodactylus* (Squamata: Gekkonidae) from northeast India. Zootaxa 4524(5): 501–535. 10.11646/zootaxa.4524.5.1 30486096

[B5] Annandale NE (1913) The Indian geckos of the genus *Gymnodactylus*. Records of the Indian Museum 9: 309–326. 10.26515/rzsi/v9/i4/1913/163665

[B6] Bharali M, Thaosen K, Vabeiryureilai M, Lalremsanga HT, Purkayatha J, Bhattacharjee R, Das M, Bohra SC (2025) A new species of *Cyrtodactylus* Gray, 1827 (Reptilia: Squamata: Gekkonidae) from the montane forests of Dima Hasao District, Assam, India. Journal of Asia-Pacific Biodiversity. 10.1016/j.japb.2025.06.008

[B7] Bhardwaj VK, Purkayastha J, Lalremsanga HT, Mirza ZA (2025) Two new species of bent-toed geckos of the genus *Cyrtodactylus* Gray, 1827 from the western Himalayas. Zootaxa 5665(2): 205–222. 10.11646/zootaxa.5665.2.3 41119793

[B8] Bhattarai S, Gautam B, Neupane BP, Khandekar A, Thackeray T, Agarwal I, Olson AR, Hogan F, Wright W (2025a) Description of two new species of *Cyrtodactylus* Gray, 1827 (Squamata, Gekkonidae) from Nepal. ZooKeys 1253: 131–160. 10.3897/zookeys.1253.161933 PMC12485482 41041656

[B9] Bhattarai S, Gautam B, Neupane BP, Khandekar A, Thackeray T, Agarwal I, Tillack F, Olson AR, Hogan F, Wright W (2025b) A review of the genus *Cyrtodactylus* Gray 1827 (Squamata: Gekkonidae) of Nepal with descriptions of three new species. Zootaxa 5594(3): 401–451. 10.11646/zootaxa.5594.3.1 40173729

[B10] Bohra SC, Zonunsanga HT, Das M, Purkayastha J, Biakzuala L, Lalremsanga HT (2022) Morphological and molecular phylogenetic data reveal another new species of bent-toed gecko (*Cyrtodactylus* Gray: Squamata: Gekkonidae) from Mizoram, India. Journal of Natural History 56(41–44): 1585–1608. 10.1080/00222933.2022.2119178

[B11] Boruah B, Narayanan S, Aravind NA, Lalronunga S, Deepak V, Das A (2024) Description of six new species of *Cyrtodactylus* Gray (Squamata: Gekkonidae) from northeastern India. Vertebrate Zoology 74: 453–486. 10.3897/vz.74.e124752

[B12] Chan KO, Grismer LL (2022) GroupStruct: An R package for allometric size correction. Zootaxa 5124(4): 471–482. 10.11646/zootaxa.5124.4.4 35391110

[B13] Das I, Dattagupta B, Gayen NC (1998) History and catalogue of reptile types in the collection of the Zoological Survey of India. Journal of South Asian Natural History 3: 121–172.

[B14] Grismer LL, Wood PL, Poyarkov NA, Le MD, Kraus F, Agarwal I, Oliver PM, Nguyen SN, Nguyen TQ, Karunarathna S, Welton LJ, Stuart BL, Luu VQ, Bauer AM, O’Connell KA, Quah ESH, Chan KO, Ziegler T, Ngo H, Nazarov RA, Aowphol A, Chomdej S, Suwannapoom C, Siler CD, Anuar S, Tri NV, Grismer JL (2021) Phylogenetic partitioning of the third-largest vertebrate genus in the world, *Cyrtodactylus* Gray, 1827 (Reptilia; Squamata; Gekkonidae) and its relevance to taxonomy and conservation. Vertebrate Zoology 71: 101–154. 10.3897/vz.71.e59307

[B15] Kamei RG, Mahony S (2021) A new species of Bent-toed gecko (Squamata: Gekkonidae: *Cyrtodactylus* Gray, 1827) from the Garo Hills, Meghalaya State, north-east India, and discussion of morphological variation for *C. urbanus*. Herpetological Journal 31: 177–196. 10.33256/31.3.177196

[B16] Kumar S, Stecher G, Suleski M, Sanderford M, Sharma S, Tamura K (2024) MEGA12: Molecular Evolutionary Genetics Analysis Version 12 for adaptive and green computing. Molecular Biology and Evolution 41: 1–9. 10.1093/molbev/msae263 PMC11683415 39708372

[B17] Lalremsanga HT, Chinliansiama H, Bohra SC, Biakzuala L, Vabeiryureilai M, Muansanga L, Malsawmdawngliana F, Hmar GZ, Decemson HT, Siammawii V, Das M, Purkayastha J (2022) A new bent-toed gecko (*Cyrtodactylus* Gray: Squamata: Gekkonidae) from the state of Mizoram, India. Zootaxa 5093(4): 465–482. 10.11646/zootaxa.5093.4.5 35391474

[B18] Lalremsanga HT, Colney Z, Vabeiryureilai M, Malsawmdawngliana F, Bohra SC, Biakzuala L, Muansanga L, Das M, Purkayastha J (2023) It’s all in the name: Another new *Cyrtodactylus* Gray (Squamata: Gekkonidae) from northern Mizoram, North-east India. Zootaxa 5369(4): 553–575. 10.11646/zootaxa.5369.4.5 38220699

[B19] Leary S, Underwood W, Anthony R, Cartner S (2013) AVMA Guidelines for the Euthanasia of Animals, 2013 Edition. American Veterinary Medical Association, Schaumburg, 102 pp.

[B20] Macey JR, Larson A, Ananjeva NB, Fang Z, Papenfuss TJ (1997) Two novel gene orders and the role of light-strand replication in rearrangement of the vertebrate mitochondrial genome. Molecular Biology and Evolution 14: 91–104. 10.1093/oxfordjournals.molbev.a025706 9000757

[B21] Mahony S, Kamei RG (2022) A new species of *Cyrtodactylus* Gray (Squamata: Gekkonidae) from Manipur State, northeast India, with a critical review highlighting extensive errors in literature covering bent-toed geckos of the Indo-Burma region. Journal of Natural History 55(39–40): 2445–2480. 10.1080/00222933.2021.1994667

[B22] Mirza ZA, Bhosale H, Ansari F, Phansalkar P, Sawant M, Gowande G, Patel H (2021) A new species of geckos of the genus *Cyrtodactylus* Gray, 1827 from Arunachal Pradesh, India. Evolutionary Systematics 5: 13–23. 10.3897/evolsyst.5.61667

[B23] Mirza ZA, Bhosale HS, Thackeray T, Phansalkar P, Sawant M, Gowande GG, Patel H (2022) A new species of bent-toed geckos of the genus *Cyrtodactylus* Gray, 1827 from western Arunachal Pradesh, India. Herpetozoa 35: 65–76. 10.3897/herpetozoa.35.e80610

[B24] Mohapatra PP, Ray S, Bhupathy B, Deuti K, Sethy PGS (2025) Checklist of Fauna of India: Reptilia. Version 2.0. Zoological Survey India. 10.26515/Fauna/2/2025/Chordata:Reptilia

[B25] Nguyen L, Schmidt HA, von Haeseler A, Minh BQ (2015) IQ-TREE: A Fast and effective stochastic algorithm for estimating maxi­mum-likelihood phylogenies. Molecular Biology and Evolution 32(1): 268–274. 10.1093/molbev/msu300 PMC4271533 25371430

[B26] Purkayastha J, Das M, Bohra SC, Bauer AM, Agarwal I (2020) Another new *Cyrtodactylus* (Squamata: Gekkonidae) from Guwahati, Assam, India. Zootaxa 4732(3): 375–392. 10.11646/zootaxa.4732.3.2 32230247

[B27] Purkayastha J, Lalremsanga HT, Bohra SC, Biakzuala L, Decemson H, Muansanga L, Vabeiryureilai M, Chauhan S, Rathee YS (2021) Four new Bent-toed geckos (*Cyrtodactylus* Gray: Squamata: Gekkonidae) from northeast India. Zootaxa 4980(3): 451–489. 10.11646/zootaxa.4980.3.2 34186969

[B28] Purkayastha J, Lalremsanga HT, Litho B, Rathee YS, Bohra SC, Mathipi V, Biakzuala L, Muansanga L (2022) Two new *Cyrtodactylus* (Squamata, Gekkonidae) from Northeast India. European Journal of Taxonomy 794: 111–139. 10.5852/ejt.2022.794.1659

[B29] R Core Team (2021) R: A Language and Environment for Statistical Computing. R Foundation for Statistical Computing, Vienna, Austria. https://www.r-project.org

[B30] Thorpe RS (1975) Quantitative handling of characters useful in snake systematics with particular reference to interspecific variation in the ringed snake *Natrix natrix* (L.). Biological Journal of the Lin­nean Society 7: 27–43. 10.1111/j.1095-8312.1975.tb00732.x

[B31] Thorpe RS (1983) Phylogenetic analysis of range expansion in the grass snake: Reticulate evolution: Primary and secondary contact zones. In: Felsenstein J (Ed.) Numerical Taxonomy: Proceedings of a NATO Advanced Study Institute. NATO Advanced Study Institute Series G (Ecological Sciences), No.1, Springer, Berlin, Heidelberg, and New York, 464–468. 10.1007/978-3-642-69024-2_47

[B32] Uetz P, Freed P, Aguilar R, Reyes F, Hošek J (2026) The Reptile Database. https://reptile-database.reptarium.cz/ [accessed on 23 January 2026]

[B33] Venables WN, Ripley BD (2002) Modern Applied Statistics with S. 4^th^ Edition. Springer, New York, 498 pp. 10.1007/978-0-387-21706-2

[B34] Wickham H (2009) ggplot2: Elegant Graphics for Data Analysis. Springer, New York, 213 pp. 10.1007/978-0-387-98141-3

