# Supplementary material for: Description of a new species of Cyrtodactylus Gray, 1827 (Reptilia, Gekkonidae) from India with redescriptions of the holotypes of C. gubernatoris (Annandale, 1913) and C. himalayicus (Annandale, 1906)
Source: Zookeys. 2026 Apr 30;1278:317–38. doi: 10.3897/zookeys.1278.186655 (PMC13153811; doi:10.3897/zookeys.1278.186655)
Supplement: Supplementary material 1 — Morphometric characters [file zookeys-1278-317_article-186655__-s001.docx]

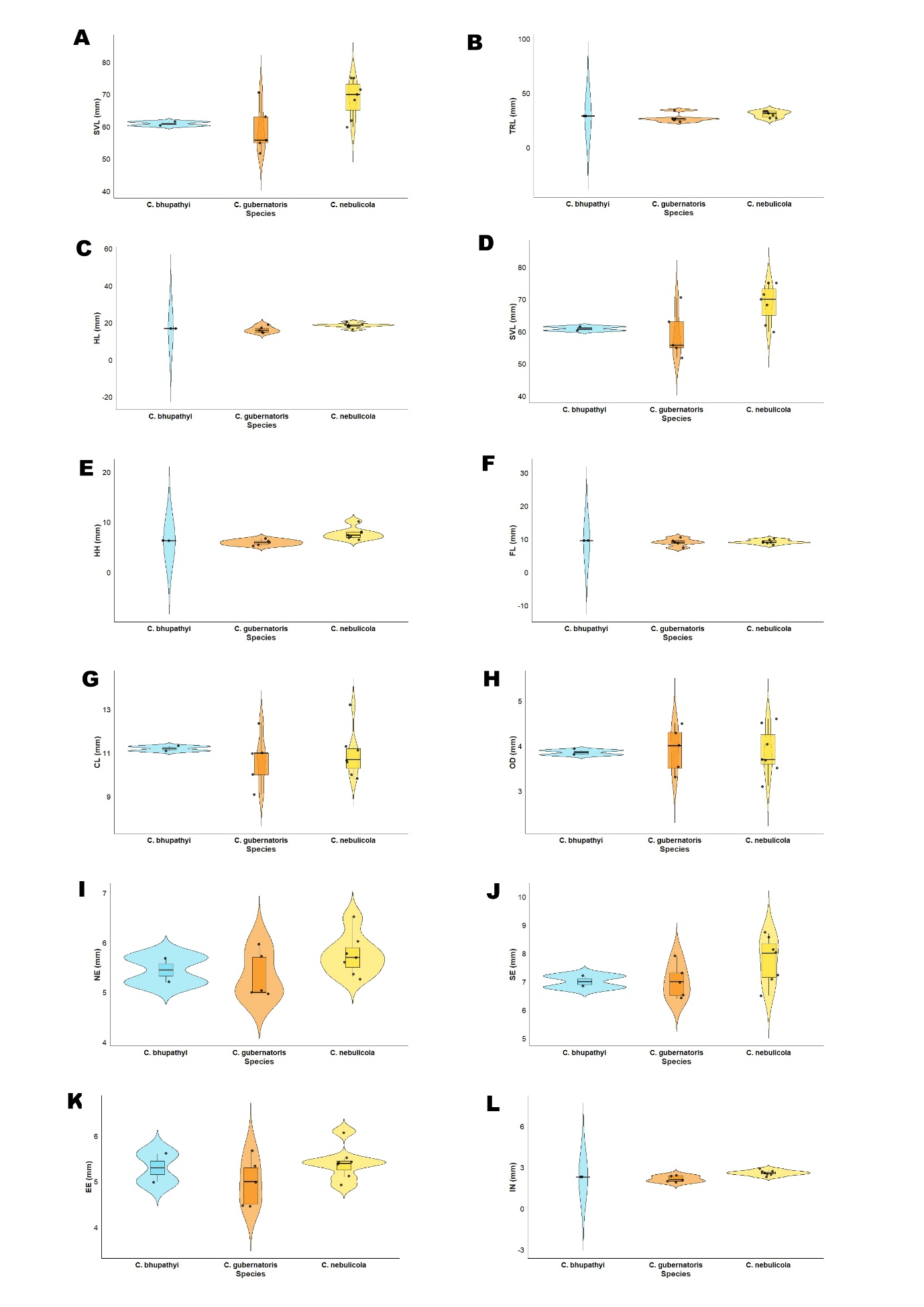


**Supplementary material Figure S1.** Violin plots of morphometric characters of three closely related members of *Cyrtodactylus nebulicola* sp. nov., *C. gubernatoris* (Annandale, 1913) and *C. bhupathyi* Agarwal, Mahony, Giri, Chaitanya & Bauer, 2018. A. SVL; B. TRL; C. HL; D. HW; E. HH; F. FL; G. CL; H. OD; I. NE; J. SE; K. EE; and L. IN.
